# Distinct Molecular Pathways Driving Thymic and Pulmonary Neuroendocrine Neoplasms: Organ of Origin Defines Molecular Identity and Yields CD44 as a Marker of Pulmonary Neuroendocrine Tumors

**DOI:** 10.1007/s12022-026-09936-w

**Published:** 2026-10-09

**Authors:** Hanibal Bohnenberger, Stefan Küffer, Arber Qoku, Julius Bodemeyer, Nelia Nause, Björn Häupl, Kirsten Reuter-Jessen, Tessa Rosenthal, Linja Feldmann, Katharina Ofner, Daniela Frances, Celia Vogel, Christof Lenz, Moritz Jesinghaus, Omar Elakad, Denise Müller, Alexander Otto König, Tobias R. Overbeck, Alexander von Hammerstein-Equord, Marc Hinterthaner, Stefan Welter, Henning Urlaub, Florian Buettner, Thomas Oellerich, Alexander Marx, Philipp Ströbel

**Affiliations:** 1 Institute of Pathology, University Medical Center Göttingen, Robert-Koch-Str. 40, Göttingen, D-37075 Germany; 2 Goethe University Frankfurt, Frankfurt am Main, Germany; 3 Institute of Informatics, Goethe University Frankfurt, Frankfurt am Main, Germany; 4 Frankfurt Cancer Institute (FCI), Frankfurt am Main, Germany; 5 Department of Medicine, Goethe University, Frankfurt, Germany; 6 Comprehensive Cancer Center Göttingen (G-CCC), University Medical Center Göttingen, Göttingen, Germany; 7 Department of Medicine II, Hematology/Oncology, Goethe University, Frankfurt, Germany; 8 Discovery Life Sciences Biomarker Services GmbH DE, Kassel, Germany; 9 Bioanalytical Mass Spectrometry Group, Max Planck Institute for Biophysical Chemistry, Göttingen, Germany; 10 Institute of Pathology, Philipps-Universität Marburg, Marburg, Germany; 11 Department of Gastroenterology, Gastrointestinal Oncology and Endocrinology, University Medical Center, Göttingen, Germany; 12 Department of Hematology and Medical Oncology, University Medical Center Göttingen, Göttingen, Germany; 13 Department of Thoracic and Cardiovascular Surgery, University Medical Center, Georg-August University, Göttingen, Germany; 14 Department of Thoracic Surgery, Lungenklinik Hemer, Hemer, Germany

**Keywords:** Thymic neuroendocrine neoplasms, Pulmonary neuroendocrine neoplasms, Pathology, Multi-omics, Proteomics

## Abstract

Thymic and pulmonary neuroendocrine neoplasms (NEN) are morphologically similar but arise from different embryonic tissues. Whether this similarity extends to shared molecular programs is unclear. We compared 45 thymic and 37 pulmonary NEN by immunohistochemistry, targeted and exome sequencing, NanoString RNA profiling and mass spectrometry-based proteomics. The RNA and proteomic profiles of pulmonary and thymic neuroendocrine tumors (LuNET and TNET) were clearly different, while the profiles of neuroendocrine carcinomas of both organs (LuNEC and TNEC) were largely overlapping. A subgroup of TNET corresponding to NET G3 clustered with lower-grade thymic NET rather than NEC. NET of both organs showed a low mutational burden and were proliferatively quiescent but diverged metabolically: LuNET showed broad metabolic and stress signaling and TNET a quiescent phenotype with PI3K/AKT/mTOR as the only enriched pathway. NEC of both organs converged on cell cycle activation and metabolic depletion with frequent *TP53* and *RB1* co-inactivation, but diverged immunologically: LuNEC were immune-hot, whereas TNEC showed epithelial–mesenchymal transition and a markedly lower mutational burden. CD44 stood out in both the transcriptomic and the proteomic analysis and was therefore immunohistochemically validated in an independent, larger cohort that included also gastrointestinal and pancreatic tumors. CD44 was high in pulmonary NET and low in every other subgroup (*p* = 2.1 × 10⁻¹³), identifying it as a new complementary marker of pulmonary origin. In summary, thoracic NEN segregate by anatomical origin as well as by differentiation grade. Wether these differences require treatments adapted to the organ of origin remains to be established.

**Supplementary Information:**

The online version contains supplementary material available at https://doi.org/10.1007/s12022-026-09936-w.

## Introduction

**Fig. 1:**
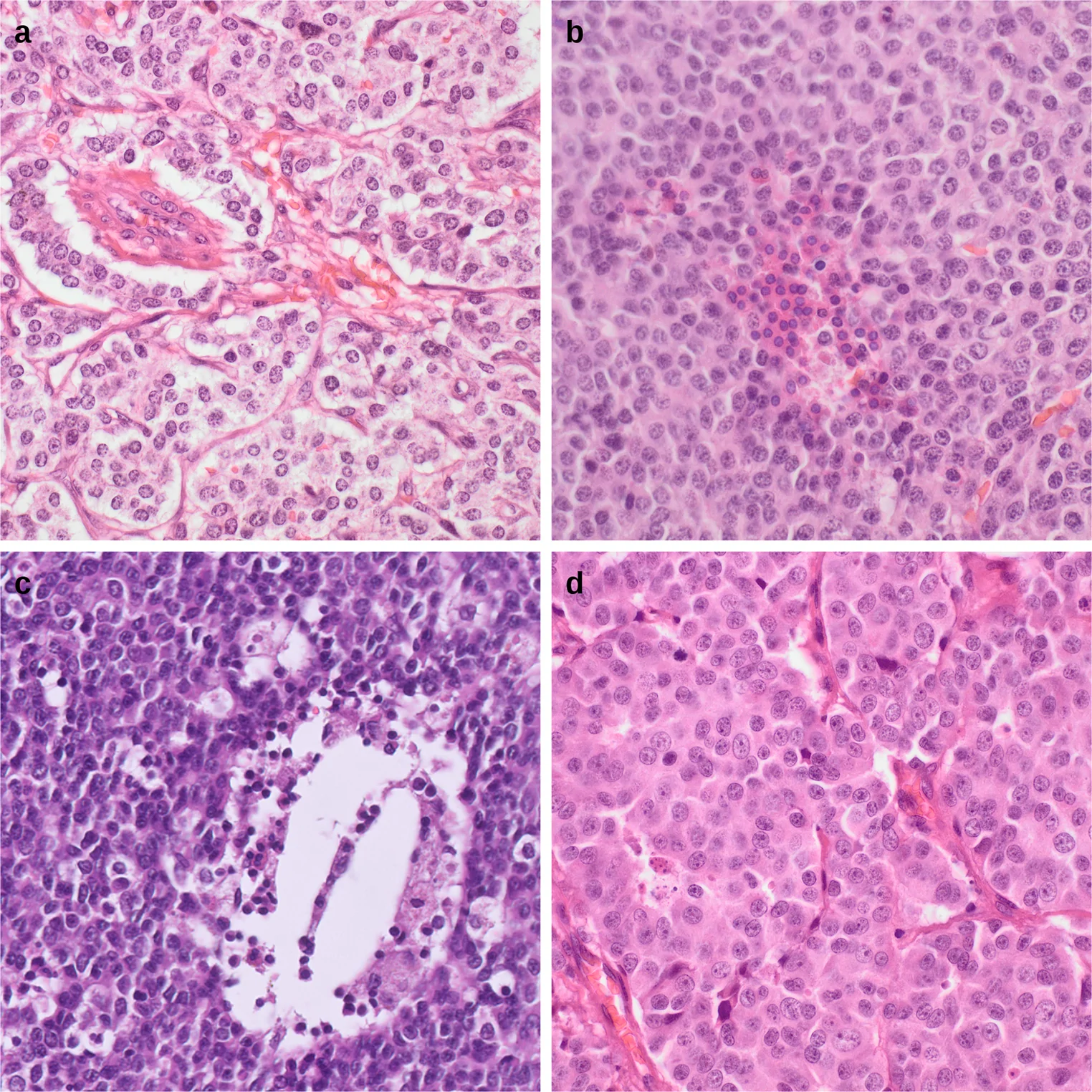
Histopathological features of thymic neuroendocrine neoplasms (haematoxylin and eosin, original magnification × 200). **a** Thymic neuroendocrine tumor (TNET) G1 (typical carcinoid) with classic organoid and nested growth and prominent vascularization. **b** TNET G2 (atypical carcinoid) with a subtle focus of tumor necrosis. c TNET G3 with central comedo-type necrosis. d Large cell neuroendocrine carcinoma (LCNEC) with solid sheets of tumor cells, frequent apoptotic bodies and numerous mitotic figures. The four panels illustrate the morphological continuum on which the classification is based: organoid architecture is retained in TNET G1-G3 but lost in LCNEC, whereas proliferative activity and necrosis increase progressively

Neuroendocrine neoplasms (NEN) of the thymus and lungs are morphologically similar and classified using the same WHO criteria [1] (Fig. 1). The revised classification has adopted elements of the common classification framework for NEN [2], which separates neuroendocrine neoplasms into neuroendocrine tumors (NET G1-G3) and neuroendocrine carcinomas (NEC), comprising large cell neuroendocrine carcinoma (LCNEC) and small cell neuroendocrine carcinoma (SCNEC). Throughout this article we use the dual nomenclature of the recent IASLC position paper [3], which retains “carcinoid” as the primary diagnostic term while adopting NET terminology: typical carcinoid (NET G1) and atypical carcinoid (NET G2), abbreviated thereafter for tumors of the lung (LuNET) and thymus (TNET). The same paper formally recognizes carcinoid/NET G3, a concept further supported by recent series applying a three-tiered, Ki-67-based grading to pulmonary NET [4, 5].

Published molecular data from both lung and thymus categorize thoracic NEN into three primary clusters enriched for NET G1, NET G2 and NEC [6, 7]. In both organs the spectrum of NET comprises a distinct additional subtype of intermediate malignancy. Such tumors were first characterized in the lung, where seven of 244 resected pulmonary NEN were well differentiated yet showed a Ki-67 index above 20%, lacked p53 and Rb1 alterations, and had an outcome intermediate between low-grade NET and high-grade NEC [8]; an analogous subtype was subsequently designated NET G3 in the thymus [7]. In the current 5th edition of the WHO classification the pulmonary counterparts are still subsumed under “atypical carcinoid with elevated mitotic counts and/or Ki-67 index” [9], but carcinoid/NET G3 has since been formally recognized for the lung [3], and a data-driven three-tier stratification of 483 pulmonary carcinoids confirmed a corresponding group in which Ki-67 was the strongest discriminator [4]. In the thymus, the corresponding subtype is characterized by carcinoid morphology, low genomic instability, a lower proliferation rate, preservation of chromogranin expression and absence of EZH2 expression [7]. These and other observations have shown that purely morphology-based grading fails to capture the molecular diversity of thoracic NEN [10].

It is currently unknown whether the morphological similarities between thymic and pulmonary NEN extend to molecular features and tumor cell biology. The two organs are derived from different embryonic structures: the thymus arises from the endoderm of the third pharyngeal pouch, which also generates the parathyroid glands [11, 12], whereas the lungs represent outpouchings of the ventral wall of the primitive foregut [13, 14]. It is therefore plausible that tumors in these organs evolve along different molecular pathways, and epidemiology supports this: TNET G2 and G1 account for most thymic cases with a marked male predominance, whereas in the lung SCNEC accounts for roughly 80% of all NEN and LuNET G2 are distinctly rare. Smoking has not been identified as a risk factor for thymic NEC.

In contrast to LuNEN, the mutational landscape of TNEN remains poorly characterized. LuNET show recurrent mutations of *MEN1*, *ARID1A*, *EIF1AX*, *ATM*, *PSIP1*, *ROBO1*, and subunits of the SWI/SNF complex [6, 15]. Pulmonary neuroendocrine carcinomas differ fundamentally in their genomic makeup: LCNEC segregate into an SCLC-like subset characterized by co-inactivation of *TP53* and *RB1* and an NSCLC-like subset carrying *STK11*, *KEAP1* and *KRAS* alterations [16], whereas small cell lung carcinoma is characterized by near-universal biallelic inactivation of *TP53* and *RB1* [17]. For thymic neuroendocrine carcinomas, by contrast, no comparable molecular data have been published to date.

The present study addresses the central question whether thymic and pulmonary NET and NEC, despite their virtually identical morphology, differ at the molecular and proteomic level. We hypothesized that the distinct embryological origins of thymus and lung are reflected in distinct molecular programs. To address this, a series of thymic and pulmonary NEN was compared using proteomic and gene expression analyses as well as comprehensive next-generation sequencing. All comparisons were structured along two axes: organ of origin (thymus versus lung) and degree of differentiation (NET versus NEC). Comparisons with neuroendocrine neoplasms of the digestive tract were restricted to the immunohistochemical validation of CD44 and served to place the thoracic findings in a wider context.

## Materials and Methods

For this study, we used archival formalin-fixed, paraffin-embedded (FFPE) tissue slides or blocks of *n* = 45 TNEN and *n* = 37 LuNEN (see Table 1) classified according to the current WHO system [1]. Large parts of the TNEN series had been characterized previously [7, 18]. To determine CD44 expression across NEN from different organs, we used multi-tissue arrays comprising 230 evaluable cases: 95 gastrointestinal and pancreatic (81 NET, 14 NEC), 117 pulmonary (67 NET, 50 NEC) and 18 thymic (14 NET, 4 NEC) NEN.

**Table 1:** Patient and tumor sample characteristics

Characteristics	TNEN (*n* = 45)	LuNEN (*n* = 37)
Median age (years, range)	60.5 (25–100)	63.5 (30–79)
Age < 60 years (n)	20	12
Age ≥ 60 years (n)	25	26
Gender		
Female (n)	8	21
Male (n)	37	17
Grading (n)		
NET G1	8	7
NET G2	22	20
NET G3	7	0
LCNEC	5	9
SCNEC	3	2

### Classification and Grading

All tumors were classified according to the proposed IASLC nomenclature [3–5] applying both morphological and immunohistochemical criteria (typical carcinoid/NET G1: differentiated NEN with < 2 mitoses per 2 mm2 and Ki-67 < 5% and no necrosis, atypical carcinoid/NET G2: differentiated NEN with 2–10 mitoses per 2 mm2 and/or Ki-67 5–20% and/or necrosis, LCNEC: high-grade non-small cell carcinomas with neuroendocrine morphology and a mitotic count of > 10 mitoses/2 mm^2^, expressing one or more neuroendocrine immunohistochemical markers, and SCNEC: small cells with scant cytoplasm, finely granular nuclear chromatin, and absent or inconspicuous nucleoli, with a high mitotic count and frequent necrosis). All thymic NET G3 showed > 10 mitoses per 2 mm^2^ and/or Ki-67 > 20% and were identified by applying the criteria established previously [7]: carcinoid morphology, retained chromogranin A expression, lacked EZH2 expression, and showed neither *TP53* nor *RB1* alterations together with a low tumor mutational burden on targeted or exome sequencing. All thymic tumors meeting the morphological criteria for LCNEC (*n* = 13) were sequenced, so that the distinction between NET G3 and LCNEC could be made on molecular grounds in every case. We note explicitly that this designation refers to the well-differentiated subgroup identified within morphologically defined LCNEC and is not equivalent to the “Thy-NET G3” tier of the morphomolecular classification proposed in the same publication, which denotes the highest-grade category.

The designations NET G1 and NET G2 are used throughout as synonyms for typical and atypical carcinoids. No pulmonary carcinoid/NET G3 were identified in this series (see Limitations).

Because of the rarity of these tumors, case collection and the individual analytical steps extended over several months, so that the number of cases available differed between assays. All 37 pulmonary cases and 38 of the 45 thymic cases were available for NanoString RNA profiling and proteomic analysis; of the seven thymic cases not included, two failed NanoString quality control and five had insufficient residual tissue, several of these being small biopsy specimens. Targeted or exome-based sequencing was performed on 19 pulmonary and 26 thymic cases. The distribution of cases across assays and diagnostic categories is given in Supplementary Table S1.

### Immunohistochemistry

Immunohistochemistry was performed on a Dako Autostainer platform with Dako EnV FLEX Peroxidase detection, using a CD44 antibody (Sigma-Aldrich, 1:1000). Staining was quantified as H-score, the sum of the percentages of tumor cells at each intensity multiplied by the respective intensity value (0–3), range 0 to 300. Pulmonary LCNEC (*n* = 18) and SCNEC (*n* = 32) showed virtually identical H-scores (median 0 in both) and were pooled as LuNEC. Raw scores are given in Supplementary Table S2, further details in Online Resource 1.

### Mass Spectrometry and Proteomic Analysis

FFPE tissue samples were processed for mass spectrometry as previously reported [19, 20]. Tumor regions containing at least 80% tumor cells were macrodissected, mixed 1:1 with a Super-SILAC standard of four neuroendocrine cell lines, processed by filter-aided sample preparation and analyzed by nano-HPLC coupled to an Orbitrap Fusion instrument. Spectra were analyzed in MaxQuant with SILAC-based quantification (Online Resource 1).

### Gene Expression Profiling

RNA was isolated from FFPE sections (RNeasy FFPE Kit, Qiagen) and 200 ng per sample analyzed on the nCounter system (NanoString) using the PanCancer Pathway Panel, covering 770 genes of 13 canonical pathways. Quality control and normalization are described in Online Resource 1.

### Next-Generation Sequencing

Targeted sequencing used the TruSight Oncology 500 (TSO500) panel (Illumina) [21], covering 523 cancer-related genes for single- and multiple-nucleotide variants, copy-number variants, microsatellite instability and tumor mutational burden (TMB). Whole-exome sequencing (*n* = 5) and the Oncomine Comprehensive Panel v3 (*n* = 1) were used for individual cases. TMB is reported for the TSO500 subcohort only; pipeline details are given in Online Resource 1.

### Bioinformatic and Statistical Analyses

Preprocessing, quality control and normalization of transcriptomic and proteomic data are described in Online Resource 1. Dimensionality reduction was performed by principal component analysis and t-distributed stochastic neighbor embedding (t-SNE). Differential expression between predefined subgroups was assessed by Welch’s two-sample t-test with Benjamini-Hochberg correction, effect sizes as Cohen’s d. CD44 H-scores were compared by Kruskal-Wallis test followed by two-sided Mann-Whitney U tests with Holm correction; all pairwise p-values reported are Holm-adjusted, and effect sizes are given as rank-biserial correlations (r). The diagnostic performance of CD44 was assessed by the area under the receiver operating characteristic curve (AUC). Overall survival was available for 200 of the 230 cases of the validation cohort. CD44 expression was related to overall survival as a continuous variable (hazard ratio per 100 H-score units) and dichotomized at two thresholds, absence of staining (H-score 0 versus > 0) and the H-score cut-off of 60 derived from the ROC analysis for the thoracic differential diagnosis (see Results), using Kaplan-Meier estimates, log-rank tests and Cox proportional hazards regression for the whole cohort and separately for NET and NEC; across the whole cohort, models were additionally adjusted for differentiation grade, organ of origin and age.

Transcriptomic and proteomic data were integrated using MuVI, a Bayesian multi-view latent factor model that learns a joint representation across molecular layers under structured sparsity. Both views were modeled as Gaussian and embedded into 32 latent factors, 31 of them informed by Hallmark gene sets from MSigDB. Factor scores were used to compare pathway-level activity between predefined subgroups.

### Use of generative AI

Generative AI (Claude, Anthropic) was used exclusively for language editing of author-written text; no part of the content was generated by AI. All authors reviewed the final text and take full responsibility for its content.

## Results

### Distinct molecular programs in pulmonary and thymic neuroendocrine neoplasms

We compared 11 LuNEC (*n* = 2 SCNEC, *n* = 9 LCNEC) and 26 LuNET (*n* = 6 NET G1, *n* = 20 NET G2) with 7 TNEC (*n* = 2 SCNEC, *n* = 5 LCNEC) and 31 TNET (*n* = 7 NET G1, *n* = 20 NET G2, *n* = 4 NET G3) using RNA expression profiling and proteomics.

**Fig. 2:**
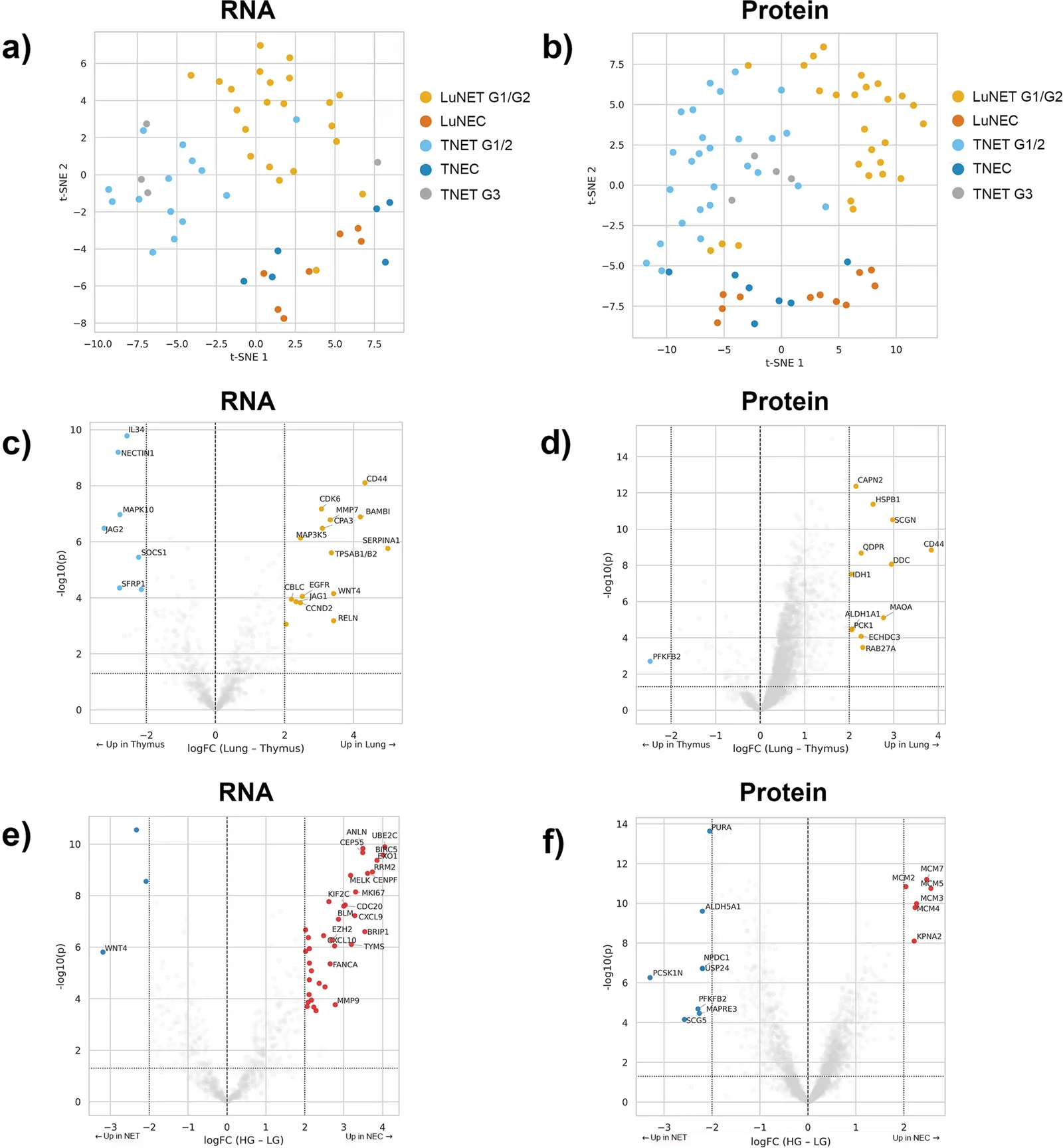
RNA and proteome profiling of pulmonary and thymic neuroendocrine neoplasms. **a**, **b** t-SNE representation of RNA expression (**a**) and proteomic (**b**) data. Both molecular layers separate the tumors by tissue of origin and by differentiation grade: pulmonary and thymic NET (LuNET, TNET) form separate clusters, whereas pulmonary and thymic NEC (LuNEC, TNEC) intermingle. Organ identity is therefore preserved in well-differentiated tumors but largely lost in carcinomas. TNET G3 cluster with TNET G1/G2 rather than with NEC. **c**,** d** Volcano plots of the most significant gene transcripts (**c**) and proteins (**d**) differentiating LuNET from TNET. **e**,** f** Volcano plots of the most significant gene transcripts (**e**) and proteins (**f**) differentiating NEC from NET. Horizontal dotted lines indicate the significance threshold, vertical dotted lines a log2 fold change of ± 2

Both RNA and protein profiles revealed distinct clusters by grade and tissue origin (Fig. 2a and b). LuNEC and TNEC were indistinguishable based on highly similar profiles, supporting shared molecular mechanisms in NEC across both organs. In contrast, LuNET and TNET exhibited characteristic separate molecular profiles. TNET G1/G2 and G3 showed significant overlap, indicating a continuous molecular transition.

**Fig. 3:**
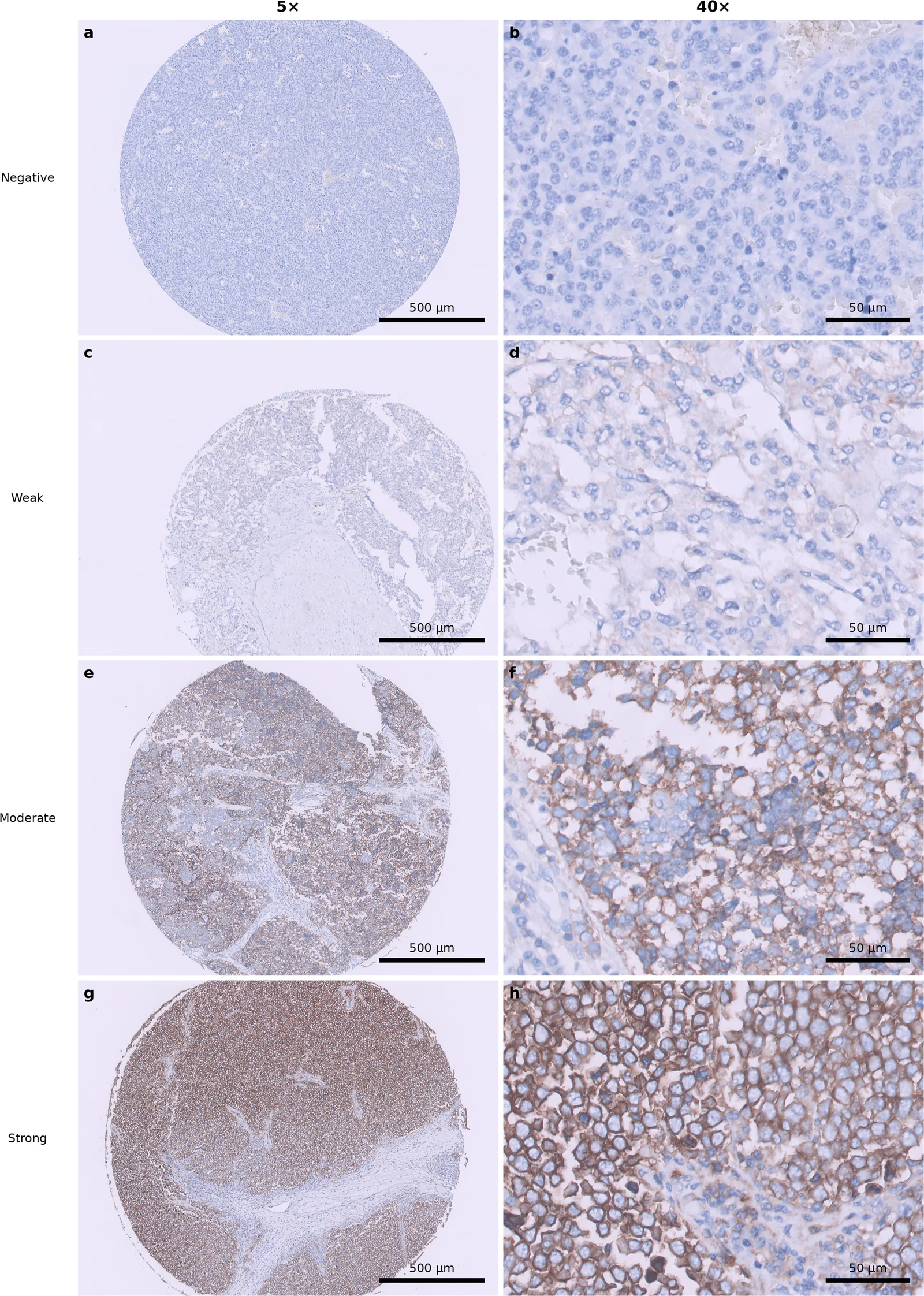
CD44 immunohistochemistry in neuroendocrine neoplasms. Representative staining intensities at low (**a**, **c**, **e**, **g**) and high (**b**, **d**, **f**, **h**) magnification: negative (**a**, **b**), weak (**c**, **d**), moderate (**e**, **f**) and strong (**g**, **h**) membranous and cytoplasmic staining, corresponding to intensity scores 0 to 3 used for H-score calculation. Scale bars 500 μm (**a**, **c**, **e**, **g**) and 50 μm (**b**, **d**, **f**, **h**)

**Fig. 4:**
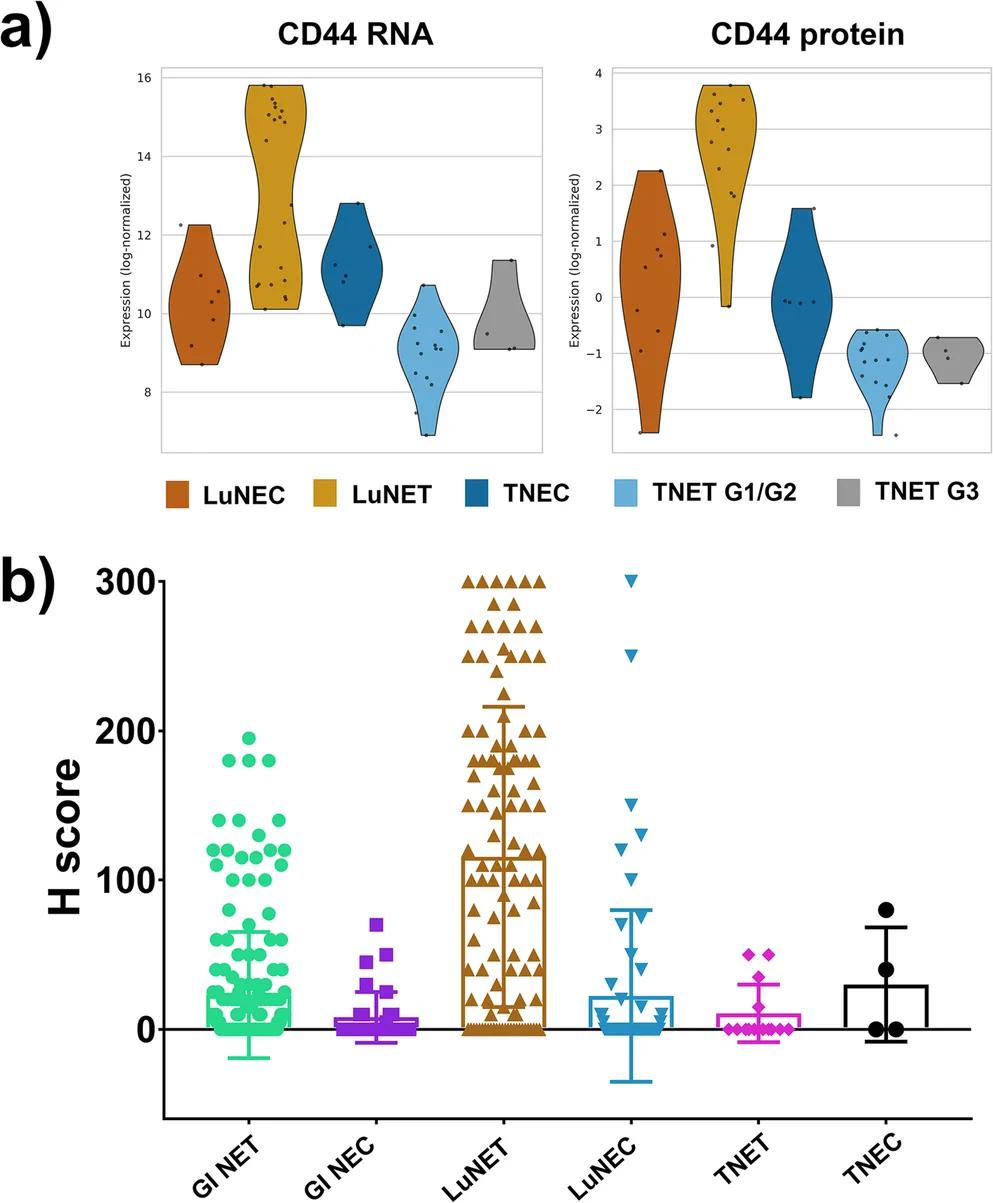
CD44 expression in thoracic and gastroenteropancreatic neuroendocrine neoplasms. **a** CD44 RNA (left) and protein (right) expression in the discovery cohort, shown as violin plots with individual cases. CD44 is highest in LuNET at both levels and was one of the few features that separate LuNET from TNET concordantly in the transcriptome and the proteome. **b** Immunohistochemical validation in an independent cohort of 230 neuroendocrine neoplasms, expressed as H-score (0-300; individual cases are shown with the median and interquartile range). CD44 expression differed across the six subgroups (Kruskal-Wallis *p* = 2.1 × 10⁻¹³) and was high only in LuNET (median 175), whereas all other groups remained low (median 0–20). The difference between NET and NEC was significant in the lung (*p* = 1.3 × 10⁻⁹) but not in the thymus or the gastrointestinal tract, indicating that CD44 reflects anatomical origin rather than differentiation grade. GI gastrointestinal and pancreatic, Lu lung, T thymic, NET neuroendocrine tumor, NEC neuroendocrine carcinoma

At the RNA level, the most significant genes distinguishing TNET (G1-G3) from LuNET (G1/G2) included *CD44*,* BAMBI*,* WNT4*,* CCND2*,* CDK6*,* CPA3*,* SERPINA1*,* MMP7*,* MAP3K5* and *EGFR* (higher in lung), as well as *NECTIN1*,* IL34*,* JAG2*,* MAPK10* and *SOCS1* (higher in thymus) (Fig. 2c). Among the most significant differentiating proteins were CD44, CAPN2, HSPB1, SCGN, DDC, QDPR, IDH1, ALDH1A1, PCK1 and MAOA (higher in lung), whereas PFKFB2 was higher in thymus (Fig. 2d). LuNET thus showed increased activity of WNT signaling, cell cycle regulation, cell adhesion and extracellular matrix remodeling together with markers of secretory and stress response, whereas TNET showed higher expression of genes involved in junctional signaling, immune and cytokine modulation and MAPK-related signaling. Among these features, CD44 was the only one that ranked among the most significant in both the transcriptomic and the proteomic analysis and for which a validated antibody suitable for FFPE material was available; it was therefore selected for immunohistochemical validation in an independent, larger cohort (Figs. 3 and 4a). Because a marker of pulmonary origin is diagnostically most relevant in neuroendocrine neoplasms of unknown primary, in which gastroenteropancreatic tumors are the principal differential diagnosis, the validation cohort comprised gastrointestinal and pancreatic (*n* = 95) in addition to pulmonary (*n* = 117) and thymic (*n* = 18) NEN.

CD44 H-scores differed markedly across the six subgroups (Kruskal-Wallis H = 68.6, df = 5, *p* = 2.1 × 10⁻¹³). Expression was high in LuNET (median 175, IQR 35–250) and low in all other groups: GI NET 10 (0–60), TNET 0 (0–11), LuNEC 0 (0–0), TNEC 20 (0–50) and GI NEC 0 (0–19). LuNET differed significantly from TNET (*p* = 1.3 × 10⁻⁴, *r* = 0.70) and from GI NET (*p* = 5.3 × 10⁻⁹, *r* = 0.57). Within the lung, NET showed significantly higher CD44 expression than NEC (*p* = 1.3 × 10⁻⁹, *r* = 0.66), whereas the corresponding differences were not significant in the thymus (*p* = 0.34, only four TNEC available) or in the gastrointestinal tract (*p* = 0.23). High CD44 expression is therefore largely restricted to pulmonary NET. Used as a marker of pulmonary NET, CD44 achieved an AUC of 0.85 against thymic NET and of 0.81 against all other subgroups combined; at an H-score cut-off of 60, sensitivity was 70% at a specificity of 94% in the thoracic differential diagnosis, that is, LuNET versus all thymic NEN (Fig. 4b).

Follow-up was available for 200 cases (135 NET, 65 NEC; 54 deaths). Across the whole cohort, higher CD44 expression was associated with longer overall survival (HR per 100 H-score units 0.57, 95% CI 0.37–0.86, *p* = 0.01; H-score ≥ 60 versus < 60: HR 0.33, 95% CI 0.16–0.70, *p* = 0.004). This association reflected the distribution of CD44 across grades: after adjustment for differentiation grade, organ and age, CD44 was no longer prognostic (H-score ≥ 60: HR 0.90, 95% CI 0.39–2.07, *p* = 0.80), whereas grade remained the dominant factor (NEC versus NET, HR 8.5, *p* < 0.001). Within NET, neither the continuous H-score (HR 0.62, 95% CI 0.32–1.19, *p* = 0.15) nor either dichotomization (H-score 0 versus > 0: HR 1.32, 95% CI 0.46–3.80, *p* = 0.60; <60 versus ≥ 60: HR 2.09, 95% CI 0.65–6.69, *p* = 0.20) was associated with survival, and the same held within NEC (continuous HR 1.15, 95% CI 0.69–1.94, *p* = 0.59; Supplementary Fig. S1). CD44 thus tracks anatomical origin without carrying prognostic information independent of grade.

In a next step, we analyzed the most significant gene transcripts and proteins responsible for the difference between pooled pulmonary and thymic NEC vs. NET (Fig. 2e **+ **f). Both datasets demonstrated a marked shift from differentiation- and metabolism-associated programs in NET toward cell-cycle, replication, and DNA repair dominance in NEC. NET showed relative enrichment of proteins and transcripts associated with metabolic regulation, vesicular transport, and cellular differentiation, including *PURA*, *ALDH5A1*, *PFKFB2*, *MAPRE3*, *SCG5*, *PCSK1N*, and *WNT4*, consistent with a more differentiated, metabolically active phenotype. In contrast, NEC showed pronounced upregulation of gene transcripts involved in cell-cycle control and genomic maintenance, including *UBE2C*, *CDC20*, *CENPF*, *RRM2*, *KIF2C*, *MELK*, *MKI67*, and *EZH2* (Fig. 2e). At the proteomic level, NEC showed increased expression of replication-associated proteins such as MCM2–MCM7 and KPNA2, supporting tight coupling of DNA synthesis, nuclear transport, and chromatin assembly [22, 23] (Fig. 2f). Both pulmonary and thymic NEC shared this transcriptional and proteomic proliferation signature, although the signal was most pronounced in LuNEC.

### The Tissue of Origin Determines the Mutational Burden in Thoracic Neuroendocrine Tumors

Sequencing was performed on 45 cases in total: 19 pulmonary (*n* = 2 SCNEC, *n* = 7 LCNEC, *n* = 3 NET G1, *n* = 7 NET G2) and 26 thymic NEN (*n* = 2 SCNEC, *n* = 3 LCNEC, *n* = 3 NET G1, *n* = 11 NET G2, *n* = 7 NET G3). Of these, 39 were analyzed with the TSO500 panel, five by whole exome sequencing (three thymic NET G2, one TNET G3, one thymic LCNEC) and one thymic NET G2 with the Oncomine Comprehensive Panel v3. In total, 228 variants were identified in LuNEC, 142 in LuNET, 61 in TNEC, 139 in TNET G1/G2, and 44 in TNET G3.

**Fig. 5:**
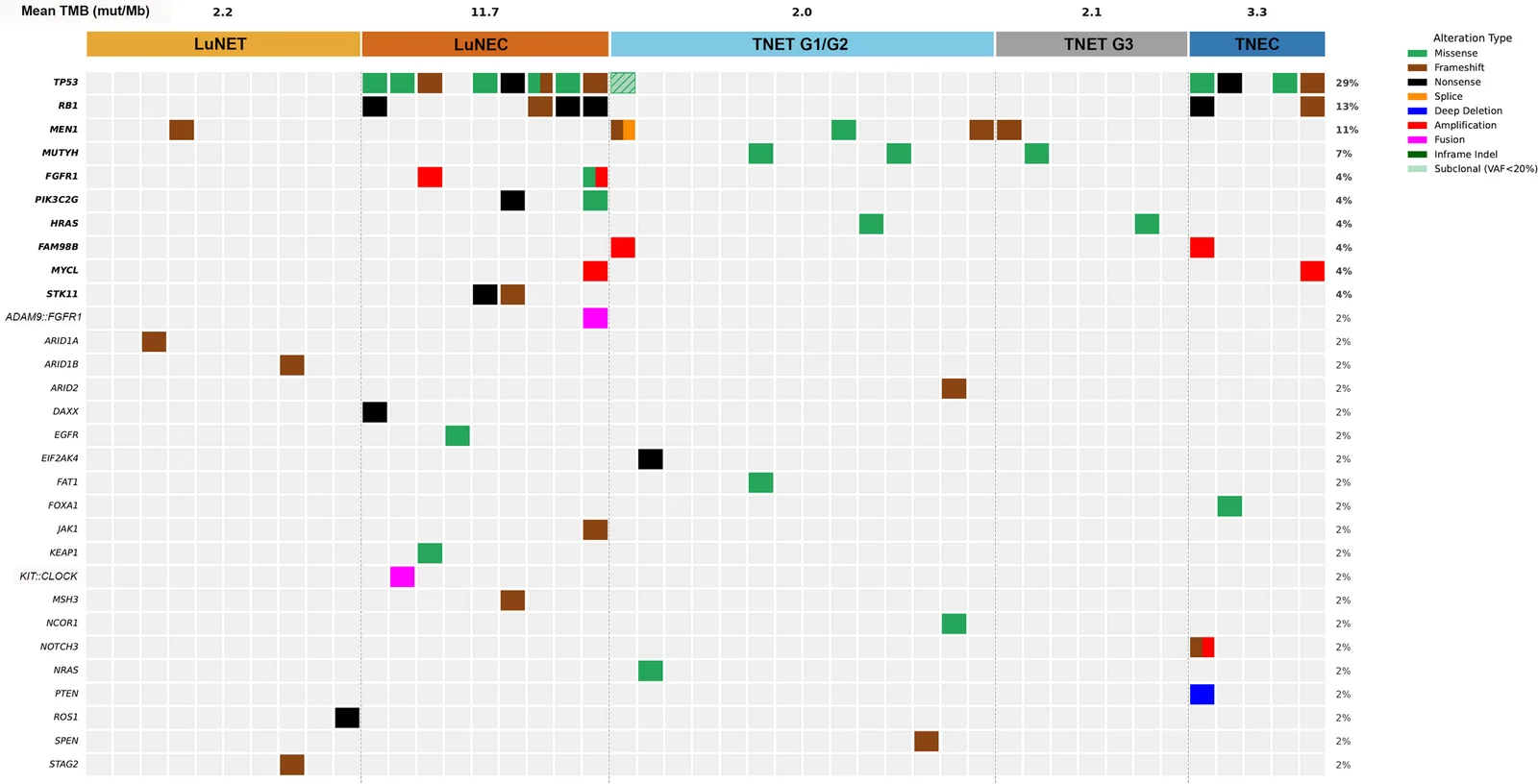
Distribution of pathogenic variants across neuroendocrine tumor subgroups. Oncoprint of pathogenic and likely pathogenic variants; each row represents a gene and each column a tumor. The top row gives the median tumor mutational burden (TMB) per subgroup, colors denote the type of alteration. The pattern illustrates the two axes of the study: alterations of *TP53* and *RB1* are essentially confined to the carcinomas of both organs, whereas *MEN1* and *MUTYH* occur across the well-differentiated tumors of thymus and lung. TNET G3 share both the low mutational burden and the absence of *TP53*/*RB1* alterations with TNET G1/G2, supporting their assignment to the well-differentiated group. All gene fusions and high-level amplifications were confined to pulmonary tumors. TMB tumor mutational burden, LuNET pulmonary NET G1/G2, LuNEC pulmonary NEC, TNET thymic NET, TNEC thymic NEC

Each variant was classified according to established clinical significance criteria. Benign and likely benign variants predominated in all subgroups, but a chi-square test of independence showed a significant difference in the distribution of variant classifications between NEC (LuNEC + TNEC) and NET (LuNET + TNET G1-G3) (χ²=18.69, df = 2, *p* = 8.7 × 10⁻⁵), with a higher proportion of pathogenic variants in the high-grade tumors. LuNEC exhibited substantially higher TMB (mean 11.7, range 6.5–16.8) than TNEC (mean 3.3, range 0–8.7) and the highest proportion of pathogenic and likely pathogenic alterations (57.1%), mainly affecting *TP53* and *RB1*. The burden was only slightly higher in TNEC than in TNET (3.3 vs. 2.0 in TNET G1/G2 and 2.1 in TNET G3). All NET displayed comparatively few pathogenic variants, including *MEN1*, *MUTYH* and *PIK3C2G*, with largely similar distributions in lung and thymus (Table 2 and Supplementary Table S3). A few cases with high-level gene amplifications (*n* = 1 LCNEC with *FGFR1*, *n* = 1 SCNEC with *FGFR1* and *MYCL*) or gene fusions (one LCNEC with *KIT::CLOCK*, one *ADAM9::FGFR1*) were all lung tumors (Fig. 5). No case showed microsatellite instability (0/39 analyzed with the TSO500 panel). Eleven of the 45 sequenced cases carried no pathogenic or likely pathogenic variant and are therefore not listed in Supplementary Table S3.

**Table 2:** Pathogenic variants across neuroendocrine tumor subgroups

Gene	LuNEC (*n* = 9)	LuNET (*n* = 10)	TNEC (*n* = 5)	TNET G1/G2 (*n* = 14)	TNET G3 (*n* = 7)	Total (*n* = 45)
TMB, mean (range)	11.7 (6.5–16.8)	2.2 (0.8–4.0)	3.3 (0–8.7)	2.0 (0–4.4)	2.1 (0–4.2)	—
Recurrently mutated genes (≥ 2 cases)						
TP53	8	0	4	1	0	13
RB1	4	0	2	0	0	6
MEN1	0	1	0	3	1	5
MUTYH	0	0	0	2	1	3
FGFR1	2	0	0	0	0	2
PIK3C2G	2	0	0	0	0	2
STK11	2	0	0	0	0	2
HRAS	0	0	0	1	1	2
MYCL	1	0	1	0	0	2
FAM98B	0	0	1	1	0	2
Selected singletons (clinically relevant)						
ARID1A	0	1	0	0	0	1
DAXX	1	0	0	0	0	1
NOTCH3	0	0	1	0	0	1
PTEN	0	0	1	0	0	1
NRAS	0	0	0	1	0	1
EIF2AK4	0	0	0	1	0	1
Structural variants / fusions						
KIT::CLOCK fusion	1	0	0	0	0	1
ADAM9::FGFR1 fusion	1	0	0	0	0	1

Complete variant data for all 45 sequenced cases are provided in Supplementary Table S1. TMB: tumor mutational burden (mut/Mb, mean and range; TSO500 subcohort). LuNEC: pulmonary NEC (SCNEC+LCNEC); LuNET: pulmonary NET G1/G2; TNEC: thymic NEC; TNET G1/G2: thymic NET G1/G2; TNET G3: thymic NET G3. Values: cases with ≥ 1 pathogenic/likely pathogenic variant. 0 (—): no pathogenic variant detected. Fusions: Archer Pan Solid Tumor panel

### Integrative Pathway Analysis Reveals Distinct Molecular Programs Across Neuroendocrine Tumor Subtypes

**Fig. 6:**
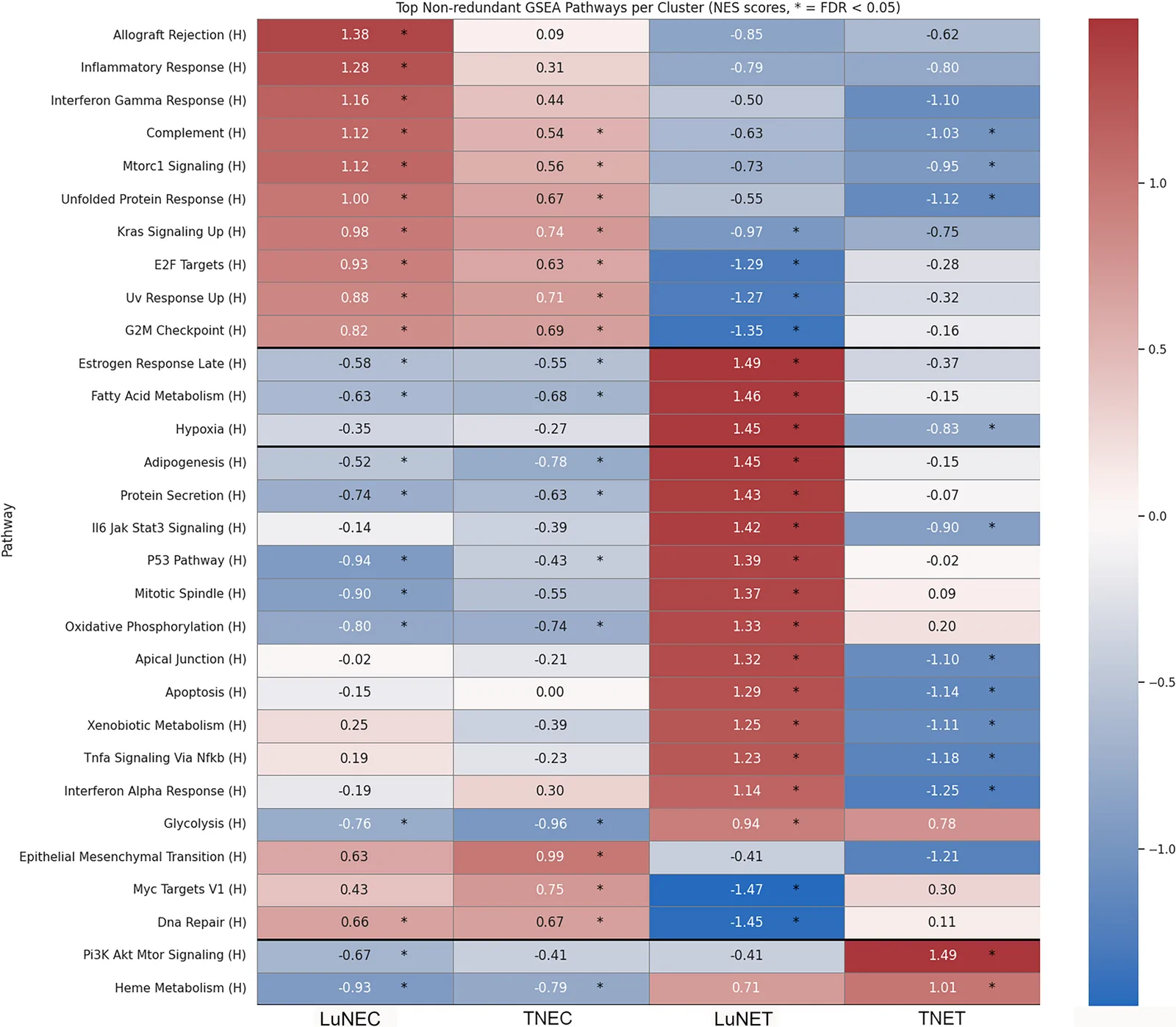
Integrated transcriptomic and proteomic analysis across subgroups of thymic and pulmonary neuroendocrine neoplasms. Pathway-level activity was derived from a joint latent factor model (MuVI) using Hallmark gene sets; values represent factor scores per subgroup. The analysis resolves the four subgroups along two biological axes. Along the differentiation axis, NEC of both organs are dominated by cell cycle, DNA replication and DNA repair programs with concomitant depletion of oxidative phosphorylation, glycolysis and fatty acid metabolism, whereas NET of both organs are proliferatively quiescent. Along the organ axis, pulmonary tumors show greater immune engagement, most pronounced in LuNEC, while thymic tumors diverge into EMT-driven invasion in TNEC and a transcriptionally quiescent, PI3K/AKT/mTOR-enriched phenotype in TNET

Integrative multi-omics analysis using MuVI delineated four transcriptionally and proteomically distinct NEN phenotypes (Fig. 6).

#### Neuroendocrine Carcinomas Share Proliferative Activation and Metabolic Depletion

Both pulmonary and thymic NEC converged on a shared molecular signature characterized by activation of canonical cell cycle programs (E2F targets, G2/M checkpoint, Myc targets V1, DNA repair) and concurrent depletion of central metabolic pathways, including oxidative phosphorylation, glycolysis and fatty acid metabolism (Fig. 6). This combination of high proliferative output with suppressed metabolic activity is consistent with a poorly differentiated phenotype in which biosynthetic resources are channeled toward cell division. In line with their mutational signatures (Fig. 5), both NEC subtypes also showed depletion of p53 pathway activity. Despite this shared architecture, LuNEC and TNEC diverged substantially in their immune and invasive profiles. LuNEC displayed the strongest immune activation of all four clusters, with enrichment of allograft rejection, inflammatory response, interferon-γ signaling and complement, consistent with an immune-hot microenvironment. TNEC showed only moderate immune activity but the highest epithelial–mesenchymal transition (EMT) activity of the entire cohort, accompanied by enrichment of protein secretion and heme metabolism. While LuNEC engages in active immune interaction, TNEC thus appears to rely on dedifferentiation and mesenchymal reprogramming, a mechanism increasingly recognized as a driver of immune evasion in thoracic malignancies [24, 25].

#### Neuroendocrine Tumors Share Proliferative Quiescence But Diverge in Metabolic and Signaling Profiles

In contrast to the NEC, pulmonary and thymic NET showed consistent depletion of cell cycle–associated pathways, most markedly in LuNET (G2/M checkpoint, E2F targets, Myc targets V1 and DNA repair), with the same direction but attenuated effect sizes in TNET (Fig. 6). This is consistent with the well-differentiated histology and low mitotic activity of these tumors. Within this shared quiescence, LuNET and TNET diverged strikingly. LuNET was defined by broad metabolic activation spanning oxidative phosphorylation, fatty acid metabolism, glycolysis and hypoxia-related programs, accompanied by stress and inflammation signaling through TNFα/NF-κB, IL-6/JAK/STAT3, p53, interferon-α and apoptosis, suggesting a metabolically versatile, microenvironment-responsive entity in which metabolic reprogramming rather than proliferative expansion dominates. TNET, by contrast, presented a transcriptionally and proteomically quiescent phenotype in which PI3K/AKT/mTOR signaling was the sole significantly enriched pathway (1.49, FDR < 0.05), identifying this axis as a candidate growth-regulatory mechanism. TNET additionally showed marked depletion of immune-related programs and suppression of EMT, apical junction and xenobiotic metabolism, delineating an immune-cold, transcriptionally restrained entity.

## Discussion

Thymic and pulmonary neuroendocrine neoplasms are morphologically similar and have been classified using the same WHO criteria across all editions of the thoracic tumor classification. However, the two organs arise from different embryonic structures, and their relative frequencies and clinical backgrounds differ substantially. The present study is the first to systematically compare the transcriptomic, proteomic and genomic profiles of a large cohort of TNEN and LuNEN across all WHO-defined subtypes including thymic NET G3, and to our knowledge the first application of mass-spectrometry-based proteomics to thymic neuroendocrine tumors.

RNA expression and protein profiles converged on distinct molecular clusters defined by tumor grade and tissue of origin. LuNET and TNET formed separate clusters at both levels, indicating that their divergence is maintained across multiple layers of gene regulation: LuNET were characterized by broad metabolic activation with stress and inflammation signaling, TNET by a transcriptionally quiescent phenotype with PI3K/AKT/mTOR as the sole significantly enriched pathway. Among the differentially expressed proteins, CD44 was validated by immunohistochemistry in an independent cohort of 230 NEN and proved to be expressed at high levels in LuNET but at low levels in thymic and gastroenteropancreatic tumors, identifying it as a correlate of anatomical origin rather than of differentiation grade. Consistent with this, CD44 status was not associated with survival within NET or within NEC, and the apparent prognostic effect across the whole cohort was fully accounted for by grade.

The diagnostic implications require careful framing. CD44 is a transmembrane glycoprotein involved in cell adhesion, matrix remodeling and stemness signaling, and its higher expression in LuNET is consistent with the enrichment of extracellular matrix and WNT-related programs in these tumors. It is not a lineage marker, and its value must be judged against the markers currently used to assign the site of origin. OTP is the most specific of these, with nuclear expression in approximately 80% of pulmonary carcinoids and virtual absence from other tumor types (sensitivity 80.2%, specificity 99.4%) [26], so that a positive result is close to conclusive while roughly one fifth of pulmonary carcinoids remain unrecognized. PAX8 has conversely been proposed as a marker of thymic origin, but its sensitivity is limited: it was positive in 8 of 25 thymic versus 2 of 25 pulmonary neuroendocrine tumors [27], and in our own series of 45 TNEN in 21 cases (47%), with TTF-1 negative in all but two LCNEC (Supplementary Table S4) [28]. A substantial proportion of cases is therefore negative for both markers, and it is in this constellation that an additional discriminator would be of value. CD44 separated LuNET from thymic NEN with an AUC of 0.85, reaching 94% specificity at 70% sensitivity at an H-score threshold of 60. These figures are inferior to those reported for OTP, and CD44 was not tested head-to-head against OTP or PAX8 on the same cases; it should therefore be regarded as complementary and subject to further prospective evaluation.

NEC from both organs clustered together in transcriptomic and proteomic analyses, indicating convergent molecular programs despite different tissues of origin. The shared signature was dominated by cell-cycle and DNA replication pathways, including upregulation of *MCM2–7*, and accompanied by a high rate of concurrent T*P53* and *RB1* inactivation (6 of 14 NEC cases, 43%). MCM2–7 forms the core of the eukaryotic replicative helicase, and its abundance in proliferating cells buffers replication stress [22, 23]. Elevated MCM expression has been reported in neuroendocrine tumors of the prostate [29], the gastroenteropancreatic tract [30, 31] and in neuroblastoma [32], correlating with poor survival and metastatic potential; therapeutic targeting is being explored but has not reached clinical application [33]. *EZH2*, another top upregulated gene in NEC, has previously been described as a prognostic factor in pulmonary and thymic NEN and was instrumental in the immunohistochemical distinction of NET G3 from NEC [7].

Despite their shared proliferative signature, LuNEC and TNEC diverged substantially in their immune and invasive profiles. LuNEC displayed the strongest immune activation of all four clusters, consistent with an immune-hot microenvironment, whereas TNEC showed the highest epithelial–mesenchymal transition activity across the entire cohort. EMT is increasingly recognized as a mechanism of immune evasion in thoracic malignancies [24, 25], and the combination of high EMT activity with comparatively low immune engagement suggests that mesenchymal reprogramming may constitute an alternative escape strategy in thymic neuroendocrine carcinomas. Their mutational profiles differed as well: LuNEC exhibited a substantially higher tumor mutational burden (mean 11.7 mut/Mb) than TNEC (mean 3.3 mut/Mb), consistent with the association of pulmonary tumors with cigarette smoking. The mutational landscape of LuNET and TNET was characterized by few pathogenic variants, most recurrently *MEN1*, followed by *MUTYH* and *HRAS*, in good agreement with previous reports in pulmonary NET [15].

The identification of PI3K/AKT/mTOR signaling as the sole significantly enriched pathway in TNET raises the hypothesis that these tumors may be particularly dependent on this axis. While everolimus is approved for gastroenteropancreatic NET [34], evidence for thymic NET remains limited [35]. It must be emphasized, however, that this inference rests entirely on pathway-level enrichment in observational data from a small number of cases. No functional validation was performed, and no clinical outcome data were available for this subcohort. The same reservation applies to the EMT-driven, immune-cold phenotype of TNEC, which is consistent with data linking mesenchymal reprogramming to poor checkpoint inhibitor response [36] but was not tested experimentally. Whether these observations have implications for site-adapted treatment decisions remains to be determined.

A central finding of this study is the further molecular characterization of thymic NET G3. In both transcriptomic and proteomic analyses, TNET G3 clustered with TNET G1/G2 rather than with NEC. Their mutational burden was comparable to that of TNET G1/G2 (mean TMB 2.0 and 2.1 mut/Mb, respectively), they lacked the *TP53 / RB1* co-mutations characteristic of NEC, and *MEN1* mutations were observed in 1 of 7 cases, further aligning this group with the lower-grade thymic NET. These findings support the proposal that thymic NET G3 represents a biologically distinct intermediate-grade subtype with increased proliferative activity but retained molecular programs of differentiated NET [7]; our proteomic data, the first available for thymic NET, provide independent multi-omics evidence that TNET G3 should not be conflated with carcinomas. Because no pulmonary carcinoid/NET G3 could be identified in our series, these conclusions apply to thymic tumors only. It is nevertheless notable that the IASLC has recently recognized carcinoid/NET G3 as a distinct category in the lung and also describes these tumors as molecularly closer to NET than to high-grade NEC [3], that a data-driven stratification of 483 pulmonary carcinoids identified a corresponding group [4], and that an earlier series characterized seven well-differentiated pulmonary NEN with a Ki-67 index above 20% lacking *p53* and *Rb1* alterations [8]. Our thymic observations therefore parallel an independent development in a different organ.

Several limitations should be considered. First, this is a retrospective study using FFPE tissue collected from multiple contributing institutions over decades, which may have affected protein and RNA quality; both resection specimens and biopsies were included, and residual material was exhausted in five thymic cases, which could therefore not be profiled. Second, sequencing was performed using three different platforms (TSO500, WES, Oncomine) with differing genomic coverage. However, the key recurrently mutated genes—*TP53*, *RB1*, *MEN1*, *HRAS*, *STK11*—are covered by all platforms, ensuring comparability of the core mutational landscape. TMB values are reported for the TSO500 subcohort only. The apparent absence of pulmonary NET G3 from our case collcetion is another methodological limitation. Fourth, the number of thymic NET G3 is small: seven cases were identified in the whole cohort, of which four had sufficient residual material for transcriptomic and proteomic profiling, although all seven were characterized by sequencing. Fifth, after subdivision into NET and NEC the pulmonary cohort is modest in size, and considerably larger molecular series of pulmonary carcinoids are available [37]; our pulmonary findings should therefore be regarded as confirmatory rather than as an independent characterization of pulmonary NET heterogeneity. Finally, all therapeutic and diagnostic implications derived here are based on observational molecular profiling without functional validation; outcome data were available only for the immunohistochemical validation cohort, and CD44 was not compared head-to-head with OTP or PAX8 on the same cases.

In conclusion, thoracic NEN segregate into molecular entities organized along two axes: differentiation grade and anatomical origin. The divergence between pulmonary and thymic NET is pronounced and is maintained across the transcriptomic and the proteomic level, whereas NEC from both organs converge on a shared proliferative program. CD44 provides a first immunohistochemical correlate of this divergence and may complement established markers of anatomical origin. The enrichment of PI3K/AKT/mTOR signaling in thymic NET and the divergent immune phenotypes of the two organs raise the possibility that treatment may need to be adapted to the organ of origin, a hypothesis that requires prospective evaluation. At the molecular level, thymic NET G3 clearly align with lower-grade thymic NET, paralleling the recent formal recognition of carcinoid/NET G3 in the lung. 

## Supplementary Information

Below is the link to the electronic supplementary material.

Supplementary Material 1

Supplementary Material 2

Supplementary Material 3

Supplementary Material 4

Supplementary Material 5

Supplementary Material 6

## Data Availability

All data supporting the findings of this study are available within the paper and its Supplementary Information.
