# Supplementary material for: Distinct Molecular Pathways Driving Thymic and Pulmonary Neuroendocrine Neoplasms: Organ of Origin Defines Molecular Identity and Yields CD44 as a Marker of Pulmonary Neuroendocrine Tumors
Source: Endocr Pathol. 2026 Oct 9;37(1):40. doi: 10.1007/s12022-026-09936-w (PMC13652782; doi:10.1007/s12022-026-09936-w)
Supplement: Supplementary file 1 [file 12022_2026_9936_MOESM5_ESM.docx]

# Online Resource 1

Distinct Molecular Pathways Driving Thymic and Pulmonary Neuroendocrine Neoplasms: Organ of Origin Defines Molecular Identity and Yields CD44 as a Marker of Pulmonary Neuroendocrine Tumors

Hanibal Bohnenberger, Stefan Küffer, Arber Qoku, Julius Bodemeyer, Nelia Nause, Björn Häupl, Kirsten Reuter-Jessen, Tessa Rosenthal, Linja Feldmann, Katharina Ofner, Daniela Frances, Celia Vogel, Christof Lenz, Omar Elakad, Denise Müller, Alexander Otto König, Tobias R. Overbeck, Alexander von Hammerstein-Equord, Marc Hinterthaner, Stefan Welter, Henning Urlaub, Florian Buettner, Thomas Oellerich, Alexander Marx, and Philipp Ströbel

## Supplementary Methods

### Immunohistochemistry — Scoring Details

Immunohistochemistry (IHC) was performed after antigen retrieval on a Dako Autostainer platform using Dako EnV FLEX Peroxidase as the detection system. All stains were performed using standard protocols. The antibody against CD44 was purchased from Sigma-Aldrich (1:1000). CD44 expression was quantified using the H-score. For each case, the percentage of tumor cells showing no (0), weak (1+), moderate (2+) or strong (3+) membranous staining was estimated, and the H-score was calculated as the sum of the percentage of cells at each intensity multiplied by the respective intensity value, giving a range from 0 to 300. Pulmonary large cell neuroendocrine carcinoma (n=18) and small cell lung carcinoma (n=32) showed virtually identical H-scores (median 0 in both groups) and were pooled as pulmonary NEC (LuNEC) for all analyses.

### Mass Spectrometry and Proteomic Analysis — Detailed Protocol

FFPE tissue samples of neuroendocrine neoplasms were processed for mass spectrometry as previously reported [12]. Briefly, tumor regions containing ≥80% tumor cells were identified on H&E-stained sections by a pathologist, and the corresponding areas from five consecutive unstained 10 µm sections were isolated by macrodissection. Tumor material was deparaffinized, resuspended in lysis buffer (100 mM ammonium bicarbonate, 4% SDS, 50 mM TCEP), incubated at 90 °C and centrifuged. Aliquots of 50 µg clarified tissue lysate were mixed 1:1 with a Super-SILAC protein standard composed of the neuroendocrine cell lines UMC11, H727, BON1, and NET18P. UMC11 and H727 were purchased from ATCC (American Type Culture Collection, Manassas, Virginia). BON1 cells were kindly provided by the Department of Gastroenterology, Gastrointestinal Oncology and Endocrinology, University Medical Center Göttingen (A. König), and NET18P cells by the ENETS Center of Excellence, University Medical Center Hamburg-Eppendorf (J. Schrader). All cell lines were metabolically labelled with heavy isotopes of arginine (Arg+10) and lysine (Lys+8). Protein mixtures were processed by filter-aided sample preparation (FASP), including urea washing, alkylation with iodoacetamide, and overnight trypsin digestion. Peptides were fractionated using the Pierce™ High pH Reversed-Phase Peptide Fractionation Kit and separated by nano-HPLC (UltiMate 3000 RSLCnano, Thermo Fisher Scientific) coupled online to an Orbitrap Fusion mass spectrometer operating in data-dependent Top Speed mode. MS/MS spectra were analyzed in MaxQuant using the Andromeda search engine against the UniProtKB/Swiss-Prot human reference proteome, with precursor and fragment ion tolerances of 6 ppm and 20 ppm, respectively, 1% FDR at peptide and protein levels, and SILAC-based quantification (Lys+0/Arg+0 and Lys+8/Arg+10).

### Gene Expression Profiling — RNA Isolation and Quality Control

Probe material was collected from 4 marked sections of 10 µm FFPE slices by scratching the areas with a scalpel. RNA was isolated according to the manufacturer’s recommendations using the “Purification of Total RNA from FFPE Tissue Sections” protocol of the RNeasy FFPE Kit (Qiagen, Hilden, Germany). The RNA was checked for quality and quantity using the NanoDrop 2000 and the 2100 Bioanalyzer RNA 6000 Nano Kit (Agilent). 200 ng RNA of each sample was hybridized for 15 hours and analyzed on the nCounter® Analysis Systems (NanoString, Seattle, USA) using the nCounter PanCancer Pathway Panel including 770 genes of 13 cancer-associated canonical pathways.

### Next-Generation Sequencing — Detailed Pipeline

Targeted sequencing was performed as previously described [13] using the TruSight Oncology 500 (TSO500) panel (Illumina, San Diego, CA, USA), covering 523 cancer-related genes to detect single- and multiple-nucleotide variants, copy-number variants, microsatellite instability, and tumor mutational burden. Libraries were prepared according to the manufacturer’s protocol and sequenced on an Illumina NextSeq 500/550 system with a paired-end 2 × 101 bp configuration. Raw BCL files were converted to FASTQ using bcl2fastq v2.20, and data processing, including alignment to the GRCh38/hg38 reference genome and variant calling was performed with the TSO500 Local App under default parameters. Variants were annotated and classified using ClinVar and OncoKB. The mean on-target coverage was approximately ≥500×, with a minimum of 100× for confident variant calling.

Sequencing was performed on 45 cases in total. Thirty-nine were analyzed with the TSO500 panel. For five thymic cases (three NET G2, one NET G3, one LCNEC), whole-exome sequencing (WES) was performed using Illumina library preparation chemistry and sequenced on an Illumina platform in paired-end mode. Reads were aligned to the GRCh38/hg38 reference genome, and variant calling and annotation were performed with the same ClinVar and OncoKB reference databases. One further thymic NET G2 was analyzed using the Oncomine Comprehensive Panel v3 (Thermo Fisher Scientific, Waltham, MA, USA). Due to differences in genomic coverage and variant-calling algorithms across platforms, tumor mutational burden (TMB) values are reported for the TSO500 subcohort only and are not directly comparable between sequencing approaches.

### Bioinformatic and Statistical Analyses — Preprocessing, Quality Control, and Dimensionality Reduction

Prior to downstream analyses, transcriptomic and proteomic data matrices were subjected to systematic preprocessing and quality control. Features with missing or empty identifiers, complete absence of signal across all samples, duplicated entries, or zero variance were removed to retain only informative molecular measurements.

**NanoString RNA expression quality control and normalization.** Sample-level quality control was performed using established control-based metrics. Library size, geometric mean expression of positive control probes, and mean expression of negative control probes were calculated per sample. Outlier detection was carried out using a robust median absolute deviation (MAD)-based approach, and samples failing both positive and negative control criteria were excluded from subsequent analyses (n = 2). RNA expression values were normalized using a two-step procedure. First, positive control normalization was applied by scaling each sample to the cohort-wide median of the geometric mean of positive controls. Second, normalization to housekeeping genes was performed using geometric means to correct for differences in RNA input and sample quality. After removal of control probes, normalized counts were converted to counts per million (CPM) and log2-transformed with a pseudocount of one to stabilize variance. The resulting expression matrix was used for all downstream transcriptomic analyses.

**Dimensionality reduction.** Following normalization, dimensionality reduction was performed separately for transcriptomic and proteomic datasets. Prior to analysis of the proteomic data, proteins with excessive missingness were excluded; only proteins quantified in at least 50% of samples were retained. Principal component analysis (PCA) was then applied independently to transcriptomic and proteomic data to assess global variance structure and sample clustering. RNA expression matrices were centered and scaled prior to PCA. For proteomics data, missing values were handled using a probabilistic PCA-based approach, allowing estimation of principal components without prior complete-case imputation. The resulting principal components were used for downstream visualization and comparative analyses. For visualization of sample relationships in low-dimensional space, nonlinear dimensionality reduction was performed using t-distributed stochastic neighbor embedding (t-SNE), using the first 20 principal components from RNA and proteomics data, respectively.

**Immunohistochemical analysis of CD44**. H-scores were compared across the six subgroups using the Kruskal-Wallis test, followed by two-sided Mann-Whitney U tests for predefined pairwise comparisons. p-values were adjusted for multiple testing using the Holm procedure; all pairwise p-values reported are Holm-adjusted. Effect sizes are given as rank-biserial correlations (r). The ability of CD44 to identify pulmonary NET was assessed by the area under the receiver operating characteristic curve (AUC), and sensitivity and specificity were calculated for a range of H-score thresholds. Raw H-scores for all 230 evaluable cases are provided in Supplementary **Table S3**.

**Differential expression analysis.** Differential expression analyses were performed separately for RNA and proteomics data by comparing predefined tumor subgroups. For each feature, group-wise differences were assessed using Welch’s two-sample t-test. Resulting p-values were adjusted for multiple testing using the Benjamini-Hochberg false discovery rate (FDR) correction. Log fold changes were calculated as differences in group means, and effect sizes were estimated using Cohen’s d. Differential expression results were visualized using volcano plots, highlighting features with both statistically significant FDR-adjusted p-values and biologically relevant effect sizes.

**Integrative multi-omics analysis (MuVI).** For integrative analysis of transcriptomic and proteomic data, we applied MuVI, a Bayesian multi-view latent factor model that enables joint representation learning across molecular layers while incorporating prior biological knowledge via structured sparsity. RNA and proteomics data were modeled as two Gaussian views and jointly embedded into a shared latent space comprising 32 latent factors. Hallmark gene sets from the Molecular Signatures Database (MSigDB) were ingested as prior information, yielding 31 informed latent factors, complemented by one uninformed factor to capture residual variation. Latent factor scores inferred by MuVI were subsequently used to assess pathway-level activity differences between predefined tumor subgroups. Differential factor activity was evaluated using group-wise statistical testing, and significantly enriched latent factors were visualized in a heatmap, highlighting pathways with distinct activity patterns across tumor groups.

**Supplementary Tables**

**Supplementary Table S1. Distribution of cases across assays and diagnostic categories.**

**Supplementary Table S2. CD44 H-scores in 230 neuroendocrine neoplasms.**

**Supplementary Table S3. Pathogenic and likely pathogenic variants across neuroendocrine tumor subgroups.**

**Supplementary Table S4. PAX8 and TTF-1 expression in 45 thymic neuroendocrine neoplasms.**

Each table is provided as a separate spreadsheet file.
